# Supplementary material for: Inter-agency collaboration to support the COVID-19 response and maintain malaria services in Guinea and Sierra Leone: A social network analysis
Source: PLoS One. 2025 Jun 16;20(6):e0325597. doi: 10.1371/journal.pone.0325597 (PMC12169549; doi:10.1371/journal.pone.0325597)
Supplement: Attachment — Submitted filename: Response to Reviewers.docx [file pone.0325597.s003.docx]

**[Title](http://www.malariajournal.com/info/instructions/default.asp#title%23title):** Inter-agency collaboration to support the COVID-19 response and maintain malaria services in Guinea and Sierra Leone: A social network analysis

_________________________________________________________

**Response to reviewers**

**REVIEWER #1:**

This is a well written manuscript and was easy to read with a nice flow. I have a few observations and questions:

**Reviewer remark:** Authors used participants and then key informants and respondents. For uniformity authors should choose one word for example use respondents all through or key informants and respondents were appropriate.

**Author response:** We thank the reviewer for this important feedback. We have now replaced the terms 'participants,' 'key informants,' and 'respondents,' which all refer to the same individuals who took part in the study, with 'respondents' to ensure consistency in the terminology used throughout the manuscript. As an example, the reviewer could look at the following section: “*Respondents were recruited to the study from 11 March to 29 March 2022 in Guinea, and from 5 March to 26 March 2022 in Sierra Leone. Respondents included representatives from both Ministries of Health, non-governmental organisations, international organisations, and bilateral and multilateral donors in both countries.*“

**Reviewer remark:** Line 137: Which language were the interviews and survey conducted? Earlier in the text authors mentioned English as the language used to interview then this line says translated to English. If this is truly a translation, authors should provide additional details on how this was done and who conducted the interviews and in which language.

**Author response:** We thank the reviewer for this remark. The interviews were conducted in French in Guinea and English in Sierra Leone. The translation into English only concerned the interviews carried out in Guinea, except for one interview conducted in English, given that Guinea is a French-speaking country. For the rest, we directly transcribed the interviews from English. We included the following sentence in the manuscript: “*In Guinea, all interviews were conducted in French (except for one interview which was conducted in English). In Sierra Leone, all interviews were conducted in English*.“

**Reviewer remark**: Line 147: Authors to please provide the number of organizations involved and how many respondents participated per organization and per country. How were these questionnaires administered- self, electronically, etc?

**Author response:** We thank the reviewer for these remarks. We have edited and added the following lines to our methods section:

*“In Guinea, we interviewed 16 organizations. In most interviews, only one person responded to our questions. However, in one interview, an additional individual from the same institution was present and contributed their views to some of the questions. In Sierra Leone, 19 organizations were interviewed, with one respondent from each organization. All the KIIs were audio-recorded. At the same time, one of the sub-team members took notes throughout the interviews.”*

*“The questionnaire pertaining to the social network survey was programmed on ODK, an open-source data collection software, and answers were collected on tablets by the interviewers.”* (line 111 to 118)

**Reviewer remark**: I was not so excited about the GSL acronym, is this a standardized acronym?

**Author response:** We thank the reviewer for their comment. Indeed, the term GSL is not common or standardized. To meet the journal’s word count requirements, we decided to use such a term. However, given the reviewer’s comment, we have now replaced the acronym with the full country names.

*__________________________________________________________________________________*

**REVIEWER #2:**

In this paper, the authors examine the role of inter-agency networks in sustaining essential health services during pandemics. Using malaria control efforts during the COVID-19 pandemic as a case study, the paper explores the network of organizations combating malaria in Guinea and Sierra Leone, the shifts in their interactions during the pandemic, and their collaboration with organizations involved in the COVID-19 response. The paper is well-conceived, well-written, and presents an interesting question.

**Reviewer remark**: In my opinion, it would be interesting for the reader if the introduction included an overview of both the number of COVID-19 notifications (if available) and malaria incidence during the study period in the countries.

**Author response:** We thank the reviewer for their suggestions. As requested, we have now added epidemiological data on COVID-19 and malaria in Guinea and Sierra Leone during the study period as follows: *“At the onset of the study in 2021, Guinea reported approximately 4.4 million cases of malaria, whereas Sierra Leone recorded 2.9 million cases. During the same year, Guinea documented 7.2 million cases of COVID-19 (an increase from 4.7 million in 2020), while Sierra Leone reported 921,000 cases (a decrease from 2.6 million cases in the previous year).”* (lines 62-66)

**Reviewer remark**: Moreover, the discussion could be strengthened by further refining suggestions on the role of pandemic networks in maintaining adequate healthcare services for infectious diseases.

**Author response:** Thank you for your remark. We have refined suggestions on the role of the network in maintaining healthcare services for infectious diseases by adding the following lines to our discussion section: *“Our findings indicate that networks around and within the health system play a pivotal role also in response coordination, as well as in sustaining essential health services during crises. Networks facilitate rapid information exchange, resource sharing, and collaborative decision-making which may mitigate service disruptions. Hence, decision makers should consider strengthening such networks prior to public health emergencies to ensure continuity of health care services during crises. Future investments should consider network development as part of emergency preparedness strategies.”*

**Reviewer remark**: A key lesson from the recent COVID-19 pandemic is that many infectious diseases, such as tuberculosis (García-García JM, COVID-19 Hampered Diagnosis of TB Infection in France, Italy, Spain and the United Kingdom. Arch Bronconeumol. 2022 Nov;58(11):783-785) were significantly impacted. It is crucial for scientific papers to contribute to addressing this issue, particularly in low-income countries, by emphasizing the need to incorporate these specific activities into future pandemic preparedness plans.

**Author response:** Thank you for this important comment. We fully agree that the COVID-19 pandemic highlighted the vulnerability of essential services for other infectious diseases, such as tuberculosis. Our study contributes to this discussion by emphasizing the role of established networks in maintaining essential health services during crises. We have added the following lines to the discussion section: *“The COVID-19 pandemic has also highlighted the vulnerability of essential services for other infectious diseases, such as tuberculosis, with disruptions reported in several high-income countries. In contrast, our study found that malaria-related services were largely maintained during the pandemic in Guinea and Sierra Leone. This highlights the role of established networks in maintaining essential health services during crises. Future pandemic preparedness plans, especially in low-income countries, should explicitly integrate the development of networks around the health system into strategies aimed at sustaining services for tuberculosis, malaria, and other infectious diseases, alongside emergency response efforts.”*
